# Clinical efficacy and safety of transarterial chemoembolization combined with targeted therapy and PD1 inhibitors in patients with advanced liver cancer

**DOI:** 10.12669/pjms.41.3.9799

**Published:** 2025-03

**Authors:** Yuanlong Zhou, Yuan Wang, Jisen Zhao, Linwei Kang, Zan Li

**Affiliations:** 1 Yuanlong Zhou, Department of Hepatobiliary Surgery, Affiliated Hospital of Hebei University, Baoding 071000, Hebei, China; 2 Yuan Wang. Department of Hepatobiliary Surgery, Affiliated Hospital of Hebei University, Baoding 071000, Hebei, China; 3 Jisen Zhao, Department of Hepatobiliary Surgery, Affiliated Hospital of Hebei University, Baoding 071000, Hebei, China; 4 Linwei Kang, Department of Hepatobiliary Surgery, Affiliated Hospital of Hebei University, Baoding 071000, Hebei, China; 5 Zan Lim, Department of Hepatobiliary Surgery, Affiliated Hospital of Hebei University, Baoding 071000, Hebei, China

**Keywords:** Transarterial Chemoembolization, Targeted Therapy, PD1 Inhibitors, Advanced Liver Cancer

## Abstract

**Objective:**

To investigate the clinical efficacy and safety of transarterial chemoembolization(TACE) combined with targeted therapy and PD1 inhibitors in patients with advanced liver cancer.

**Methods:**

This was a retrospective study. A total of 120 patients with advanced primary liver cancer admitted to Affiliated Hospital of Hebei University were randomly divided into two groups, with 60 patients in each group from May 2020 to May 2023. Patients in the control group received conventional TACE, and those in the experimental group received 200 mg camrelizumab once every 21 days and oral lenvatinib mesylate capsules once daily in addition to TACE. Compared the clinical efficacy, levels of tumor markers, T lymphocyte subsets, and adverse drug reactions after treatment and the improvement of quality of life(QOL) before and after treatment between the two groups of patients.

**Results:**

The overall response rate(ORR) was 80% in the experimental group and 62% in the control group, and the difference was statistically significant(p=0.03); the incidence of adverse reactions was 28% in the experimental group and 25% in the control group, with no significant difference between the two groups(p=0.68); the improvement rate of QOL score was significantly increased(p=0.03) and the deterioration rate was significantly decreased(p=0.01) in the experimental group compared with those in the control group, respectively.

**Conclusion:**

TACE combined with targeted therapy and PD1 inhibitors is significantly effective to improve the cellular immune function with no significant increase in the incidence of adverse reactions, making it an effective and safe treatment option for patients with liver cancer.

## INTRODUCTION

Liver cancer is the sixth most commonly diagnosed cancer and the third leading cause of cancer death worldwide.^1^ The main cause of primary liver cancer is viral hepatitis that is not ideally treated. Liver cancer is a highly malignant disease, and the choice of treatment depends on the stage of the disease and physical condition of the patients at the time of diagnosis. Surgery is the preferred treatment option for early liver cancer.^2^ However, liver cancer is often found unexpectedly during physical examinations because no characteristic clinical manifestations and even no symptoms are present in the early stage of this disease. Therefore, most patients are diagnosed with advanced liver cancer at their first visit, and the survival benefits of surgical treatment are not significant and the therapeutic effects are not ideal in these patients. Therefore, surgical resection is no longer an appropriate treatment option for most patients with advanced liver cancer, and local and systemic non-surgical treatment methods should be used.^3^

Current treatment options for patients with advanced primary liver cancer mainly include TACE. During this procedure, the femoral artery at the root of the thigh is punctured, and a fine catheter is inserted and advanced to the tumor in the liver under the guidance of fluoroscopy. Anticancer drugs and embolic agents are then injected into the tumor artery of the liver via the catheter. Commonly used drugs for TACE include fluorouracil injection, cisplatin for injection, and doxorubicin hydrochloride liposome injection.^4^ These common chemotherapy drugs can be used in TACE for the treatment of primary liver cancer. However, there are limitations in these treatments due to the deficiency of targeted effects to liver cancer cells, although certain therapeutic effects can be achieved. Therefore, discovery of scientific and rational drug combinations for TACE is concerned for the treatment of patients with liver cancer. Lenvatinib can kill cancer cells to provide ideal therapeutic effects when it is used in combination with TACE for the treatment of patients with primary liver cancer.^5^

Emerging clinical data suggest that immune checkpoint inhibitors combined with anti-angiogenic agents are rational treatment strategies for various malignant tumors.^6^ On this basis, the clinical efficacy of TACE combined with camrelizumab and lenvatinib in the treatment of primary liver cancer was evaluated in the present study, investigated the clinical efficacy and safety of transarterial chemoembolization(TACE) combined with targeted therapy and PD1 inhibitors in patients with advanced liver cancer.

## METHODS

This was a retrospective study. A total of 120 patients with advanced primary liver cancer admitted to Affiliated Hospital of Hebei University from May 2020 to May 2023 were randomly divided into experimental group(n=60) and control group(n=60) according to different treatment. There were 33 males and 27 females in the experimental group, with an average age of 53.31±6.25 years old (range 43-65 years), and 35 males and 25 females in the control group, with an average age of 53.74±6.70 years old (range 42-66 years). The ECOG scores of all patients were ≤2, indicating that their physical conditions were suitable for the treatment under study. The general data were comparable between the two groups of patients, with no significant differences (Table-I).

**Table-I:** Comparison of general data between the two groups (*χ̅±S*)(n=60).

	The experimental group	The control group	t/χ^2^	p
Male(n, %)	33(55%)	35(58%)	0.14	0.71
Age(years)	53.31±6.25	53.74±6.70	0.36	0.72
Body weight(kg)	64.23±8.31	64.60±8.13	0.54	0.56
ECOG score	0.65±0.17	0.62±0.15	0.44	0.63
Tumor diameter(cm)	5.57±1.21	5.64±1.52	0.28	0.72
Child class			0.29	0.60
A	53(88%)	51(85%)		
B	7(12%)	9(15%)		
Degree of differentiation			0.14	0.72
Highly differentiated	35(58%)	33(55%)		
Moderate differentiated	19(32%)	20(33%)		
Low differentiated	6(10%)	7(12%)		

p>0.05.

### Ethical Approval:

The study was approved by the Institutional Ethics Committee of Affiliated Hospital of Hebei University (No.: HDFY-LL-2022-050; Dated: February 21, 2022), and written informed consent was obtained from all participants.

### Inclusion criteria:

- Diagnosed as liver cancer by imaging and histopathological examinations.^7^
- Met the criteria of Barcelona-Clinic Liver Cancer Staging System (BCLC) intermediate and advanced stages.^8^
- With indications for TACE.
- Who were treatment naive.
- With complete clinical data.
- Without significant neurological or cognitive impairments and with the ability to cooperate with the study.
- Who agreed to the study protocol, signed a consent form, and did not withdrew from the study.

### Exclusion criteria:

- With serious underlying diseases such as diabetes, cardiovascular and cerebrovascular diseases.
- Who were allergic to the drugs used in the study.
- Complicated with other malignant tumors.
- Who had received other radiotherapy and chemotherapy.
- With mental and cognitive abnormalities, and unable to cooperate with the study.
- With other chronic diseases that may affect the study, such as chronic inflammatory diseases and autoimmune diseases.
- Malignant tumors in other sites of the body at the same time.
- With incomplete clinical data.

Patients in the control group received conventional TACE. Briefly, the femoral artery was punctured and a catheter was placed. Hepatic angiography was conducted, and artery supplying blood in the tumor was selected. The catheter was advanced into the target artery to infuse chemotherapy drugs. (“Gemcitabine 0.8-1 g/m^2^ was initially administered, followed by oxaliplatin 85-100 mg/m^2^ mixed with 10-3 ml lipiodol emulsion” was changed to) *Raltitrexed was administered after dilution for* perfusion chemotherapy, and the tumor blood vessels were slowly embolized with doxorubicin, oxaliplatin, lipiodol, and gelatin sponge particles, once every month, for a total of 2-3 times.

Patients in the experimental group were infused with camrelizumab 200 mg, once every 21 days and orally administered with lenvatinib mesylate capsules once a day at a dose of 8 mg for patients with a body weight of <60 kg and 12 mg for those with a body weight of ≥60 kg. The dose was adjusted according to the conditions of the patients at follow-up. The treatment was repeated every four weeks.

### Outcome Measures:

***Efficacy evaluation:*** Enhanced CT or MRI were conducted for the two groups of patients monthly after treatment, and disease control was determined according to imaging findings. Complete remission (CR) was defined as the absence of tumor, with a duration of >1 month; partial response (PR) as tumor reduction ≥50%, with a duration of >1 month; stable disease(SD) as tumor reduction<50%, or increase ≤25%, with a duration of >1 month; and progressive disease(PD) as tumor enlarged. ORR=(CR+PR) cases/total cases x 100%, and disease control rate (DCR)=(CR+PR+SD) cases/total cases x 100%^9^;

The levels of tumor markers before and one month after treatment: five ml of cubital venous blood samples were collected from patients and the levels of alpha-fetoprotein (AFP), carcinoembryonic antigen (CEA), fibroblast growth factor (FGF), and vascular endothelial growth factor (VEGF) were measured;

Comparative analysis of immune function parameters: Venous blood samples were collected before and after treatment to determine the different T lymphocyte subsets, including CD3^+^, CD4^+^, CD8^+^, and CD4^+^/CD8^+^, in the two groups of patients. Fasting in the in the morning, T-lymphocyte subsets in peripheral blood were determined by flow cytometry. And the above serum indexes were determined by enzyme-linked immunosorbent assay (ELISA). The levels were analyzed and compared between the two groups;

Adverse drug reactions: Adverse drug reactions that occurred in the two groups of patients within one month after administration, including proteinuria, decreased white blood cells, decreased red blood cells, decreased platelets, and gastrointestinal symptoms, were recorded; and (5) the QOL score: The improvement of QOL before and after treatment was observed using the ECOG score^10^, which was evaluated as improvement (score reduction ≥1 point), stable (score unchanged), and worsened (score increase ≥1 point).

### Statistical Analysis:

All data were analyzed using SPSS 23.0 software, and measurement data were presented as (*X̅*±*S*). Two independent sample t-test was used for comparison between groups, paired t-test was used for comparison within groups, and χ^2^ test was used for comparison of rates. Differences with a p value of <0.05 were considered statistically significant.

## RESULTS

Comparison of the clinical efficacy between the two groups of patients before and after treatment showed that the ORR was 80% in the experimental group, which was significantly increased compared with that in the control group (62%), and the difference was statistically significant(p=0.03) (Table-II).

**Table-II:** Comparison of the clinical efficacy between the two groups before and after treatment (*χ̅±S*)(n=60).

Outcomes	CR	RR	SD	PD	RR
The experimental group	22	26	7	5	48(80%)
The control group	15	22	13	10	37(62%)
*χ^2^*					4.88
*p*					0.03

p<0.05.

Changes in serum tumor markers in the two groups before and after treatment were shown in Table-III. No significant differences in AFP, CEA, FGF, and VEGF were observed between the experimental group and the control group before treatment (p>0.05), and the levels of these markers were significantly decreased in the experimental group compared with those in the control group after treatment, with statistical significances (p=0.00).

**Table-III:** Comparison of serum tumor markers between the two groups before and after treatment (*χ̅±S*)(n=60).

Items	Observation time	The experimental group	The control group	T	p
CEA(ng/ml)	Pre-treatment	6.47±0.72	6.55±0.63	0.65	0.52
	Post-treatment*	3.84±0.12	4.28±0.20	14.61	0.00
AFP(ng/ml)	Pre-treatment	558.95±89.46	557.64±86.85	0.08	0.94
	Post-treatment*	96.47±21.53	112.76±26.53	3.71	0.00
FGF(pg/ml)	Pre-treatment	9.36±1.13	9.49±1.32	0.45	0.66
	Post-treatment*	3.28±0.87	4.65±1.01	7.96	0.00
VEGF(pg/ml)	Pre-treatment	168.50±26.38	167.21±26.17	0.27	0.79
	Post-treatment*	92.72±12.75	103.40±13.52	4.82	0.00

* p<0.05.

Changes in T cell subsets before and after treatment in the two groups of patients (Table-IV) showed that the levels of CD3^+^ and CD4^+^ T cells were increased in the experimental group after treatment compared with those in the control group, with statistical significances(p=0.00); there was no statistically significant change in the level of CD8^+^ T cells(p=0.97); and the level of CD4^+^/CD8^+^ T cells was increased in the experimental group compared with that in the control group, and the difference was statistically significant(p=0.00).

**Table-IV:** Comparison of T cell subsets before and after treatment between the two groups(*χ̅±S*)(n=60).

Items		The experimental group	The control group	t	p
CD3+ (%)	Pre-treatment	44.53±7.12	44.90±7.20	0.28	0.78
	Post-treatment*	48.86±7.31	45.10±6.83	2.90	0.00
CD4+ (%)	Pre-treatment	26.31±4.05	26.52±4.37	0.27	0.78
	Post-treatment*	36.47±5.83	32.68±5.59	3.63	0.00
CD8+ (%)	Pre-treatment	22.42±3.17	22.16±3.24	0.44	0.65
	Post-treatment*	22.68±3.06	22.70±3.21	0.03	0.97
CD4+/CD8+	Pre-treatment	1.28±0.27	1.26±0.16	0.49	0.62
	Post-treatment*	1.84±0.32	1.57±0.28	4.92	0.00

* p<0.05.

The incidence of adverse drug reactions after treatment was 28% in the experimental group and 25% in the control group, with no statistically significant difference between the two groups (p=0.68) (Table-V). Comparison of the QOL score after treatment between the two groups showed that the improvement rate of QOL score was significantly increased (p=0.03) and the deterioration rate was significantly decreased(p=0.01) in the experimental group compared with those in the control group, respectively, with significant advantages observed in the experimental group in terms of QOL improvement (Table-VI).

**Table-V:** Comparison of adverse drug reactions between the two groups after treatment (*χ̅±S*)(n=60).

Groups	Hypertension	Fatigue	Diarrhea.	Weight loss	Arthralgia	Proteinuria	Incidence
The experimental group	2	5	3	2	3	2	17(28%)
The control group	2	4	2	3	2	2	15(25%)
*χ^2^*							0.17
*P*							0.68

p>0.05.

**Table-VI:** Comparison of QOL scores (ECOG) before and after treatment between the two groups (*χ̅±S*)(n=60).

Groups	Improved*	Stable	Worsened*
The experimental group	46(77%)	10(17%)	4(6%)
The control group	35(58%)	12(20%)	13(22%)
*χ^2^*	4.60	0.22	5.53
*P*	0.03	0.63	0.01

* p<0.05.

## DISCUSSION

The results of the present study showed that after treatment, the ORR was 80% in the experimental group and 62% in the control group, and the difference was statistically significant(P=0.03); and the levels of AFP, CEA, FGF, and VEGF were significantly decreased in the experimental group compared with that in the control group, with statistically significant differences(P=0.00), indicating that TACE combined with oral levatinib mesylate was safe and effective to improve the treatment efficacy and reduce the level of tumor markers in patients with hepatocellular carcinoma, with positive significance. Antiangiogenic drugs play an essential role in the treatment of hepatocellular carcinoma and are of extreme importance in delaying the progress of the disease and improving the prognosis. Levatinib mesylate can inhibit tumor angiogenesis by binding to tyrosine kinase ATP to inactivate tyrosine kinase and reduce the content of VEGF that is dependent on the VEGFR-2 pathway.^11^ Furthermore, levatinib mesylate also directly inhibits the proliferation of liver cancer cells, and the inhibitory effect is positively correlated to the concentration of VEGFR-2 in the cells. The underlying mechanism of action involves the inhibitory effect on the activation of the proliferation pathway and blockage of the cell cycle process.^12^

The results of the present study also showed that the immune function of T lymphocytes in patients treated with camrelizumab was significantly improved, and the levels of CD3^+^, CD4^+^, and CD4^+^/CD8^+^ T cells were significantly increased in the experimental group compared with those in the control group (P=0.00), with statistically significant differences; and after treatment, the improvement rate of QOL score was significantly increased(P=0.03) and the deterioration rate was significantly decreased(P=0.01) in the experimental group compared with those in the control group, respectively, suggesting that TACE combined with camrelizumab are more effective in improving the immune function and the QOL of patients. The reasons behind may involve the synergistic effect of the combination therapy, which can more easily reduce the tumor load.

The present study also showed that the incidence of adverse drug reactions was 28% in the experimental group and 25% in the control group, with no statistically significant difference between the two groups (P=0.68). Donne et al.^13^ have confirmed that camrelizumab can synergistically work with lenvatinib to reduce the levels of monocytes and macrophages in tumor tissues and improve the levels of T lymphocytes, playing an immunomodulatory role. Therefore, the incidence of adverse drug reactions did not increase significantly, and the treatment was well tolerated.

Liver cancer is not sensitive to chemotherapy and radiotherapy, and interventional chemoembolization is a common method for patients with advanced liver cancer due to its advantages of minimal trauma, mild side effects, and repeated use for treatment.^14^ Nouso et al.^15^ believed that despite the fact that TACE can block the blood supply pathway of tumors, it may also promote angiogenesis to a certain extent and consequently the formation of collateral circulation, resulting in incomplete necrosis of some nodules after surgery. Relevant studies^16^ have shown that the pathogenesis and progression of hepatocellular carcinoma are closely related to angiogenesis. VEGF induces vascular endothelial cell proliferation and involves in endothelial cell apoptosis inhibition, cell reconstruction, and protease expression to generate new capillaries and enhance vascular permeability. Furthermore, VEGF can bind to VEGFR-2 and initiate a series of biological regulatory mechanisms to promote the proliferation and migration of endothelial cells.^17^

The development and progression of malignant tumors are closely related to immune function, especially the lymphocyte-induced cellular immunity plays an important role in the regulation of tumor-related immune processes. Cellular immunity is an immune regulation mode against tumors, and mainly involves the CD3^+^-, CD4^+^-, and CD8^+^-mediated immune responses.^18^ Chow et al.^19^ found that the change in CD4^+^/CD8^+^ T cells is inversely proportional to the diameter of the tumor lesions, and can be used as an indicator for the evaluation of immune function. PD-1 pathway is a hot topic in recent years. PD-1 can mediate the immune tolerance caused by various ways in tumor patients. PD-1 protects the liver from the damage caused by the autoimmune system and the anti-inflammatory process of the body to a certain extent. However, it may also lead to immune escape of cancer cells during the progression of the tumor. This special pattern is the reason why the disease can be controlled by immunotherapy. Camrelizumab is a PD-1 inhibitor that blocks the binding of PD-1 and PD-L1 and destroys the immunosuppressive microenvironment of the tumor to exert its anti-tumor effect.^20^

### Limitations:

It includes small sample size and short follow-up duration. Future clinical studies with an increased sample size and a longer follow-up time are needed to comprehensively evaluate the effect of this treatment regimen in patients.

## CONCLUSION

In conclusion, TACE combined with camrelizumab and lenvatinib is significantly effective in the treatment of advanced primary liver cancer to improve the immune function of T lymphocytes, reduce the levels of tumor markers, and enhance the QOL of patients, with no significant increase in adverse drug reactions, making it a safe and effective treatment option.

### Authors’ Contributions:

**YZ** and **JZ:** Carried out the studies, participated in collecting data, and drafted the manuscript, and are responsible and accountable for the accuracy or integrity of the work.

**YW, LK and ZL:** Performed the statistical analysis and participated in its design. Critical analysis.

All authors read and approved the final manuscript.
